# Dental Hints Department

**Published:** 1906-09

**Authors:** B. H. Teague

**Affiliations:** Aiken, S. C.


### DENTAL HINTS DEPARTMENT

Conducted by B. H. TEAGUE, D. D. S., Aiken, S. C., to whom all contributions to this department should be sent.

Paste can be prevented from souring by adding a little pulverized blue stone.—*Pop. Mech's.*

When choosing color for partial sets, the sample teeth should be wet or the natural teeth dried.

An empty spool bobbin makes a good handle for a double-end vulcanite file.

Strips of rubber-dam 1/4 by 2 inches—should be kept handy to compress cement and polish soft analgam fillings.

A CAUSE OF FAILURE.—Many a filling that would otherwise be a failure can be saved by grinding down an opposing cusp. This is a little point, but it can save many a filling.—*F. F. Fletcher, Dental Era.*

If we do not value our skill and time *ourselves*, our *patients* certainly will not. And this is wholly the fault of the dentist himself, *for no patient will pay more than is asked of him.—P St. C. Smith, Dom. Jour.*

TO MAKE HARD PLASTER MODELS.—Mix the plaster of Paris with one-sixth of its volume of pulverized slaked lime. After the model is dried, place it in a 10 per cent solution of zinc sulphate until it is thoroughly saturated. Remove and dry.—*Dental Era.*

ADJUSTING THE RUBBER DAM.—If the gums are washed with oil of cloves immediately before adjusting the rubber dam, the pain incident to the application of clamps or ligatures is very greatly minimized.—*E. K. Wedelstaedt, The Dentist's Magazine.*

Sensitive Dentin.—I find a saturated solution of potassium carbonate in glycerin very effective for sensitive dentin. Apply to the cavity on cotton and let remain for from ten to fifteen minutes. Non-poisonous.—*L. W. Jordan, Dental Summary.*

To Remove Tin From Plates.—Small particles of tin adhering to vulcanite plates can easily be removed by mixing mercury with enough alloy to keep it from flowing, and rubbing it over the plate under fingers.—*C. W. Siefkill, Dental Brief.*

Swaging with Moldine.—By using the moldine as a die the moldine seems to draw from every point pressing the metal right into place when using it for swaging backings on teeth, swaging matrices for gold inlays and porcelain inlays from die.—*I. K. Douglas, Dental Summary.*

Taking Impressions.—In cases that are extremely sensitive and easily nauseated I have found it useful and helpful to sponge the mouth with hydrogen dioxid, then applying a 3-100 solution of eucain to the whole palate.—*T. B. Hartzell, Texas Dental Journal.*

Silver Nitrate and Cement Fillings.—A filling of oxyphosphate of zinc placed upon a surface treated with nitrate of silver will last a great deal longer and be a great deal better mass than the same mass not having the peculiar effect it gets from this film of silver albuminate.—*W. V. B. Ames, Dental Review.*

To Carry a Small Portion of Cement.—Take a very delicate broach, and either make a little ring at the end of the broach, or bend the point at an angle of 45° or 90°—just a little bend that you would almost have to use a magnifier to see—and you will find you can carry a little globule on the end of the broach. Otherwise it would tend to run back.—*W. R. Barnes, Dental Review.*

Mummifying Root-Canal Filling.—A mummifying root-canal filling that has been recommended consists of a liquid composed of equal parts of formaldehyd and creasote, and a powder consisting of 5 parts thymol, 12 parts dry alum, and 40

parts zinc oxid. If the liquid should be cloudy a few drops of alcohol will clear it.—*Stomatologist.*

The blue color so often desired in a cement filling may be obtained by using a German silver spatula—in mixing, enough of the metal wears off and is incorporated to secure the effect.

A dressing for putrescent teeth.

Phenol cryst ..............................
Thymol ..................................aa ℥ ij.
Camphor ................................ʒj.

Melt together by gently heating in a dry test-tube.—*Era.*

If you just will use your rubber dam more than once wash it with Parke Davis & Co.'s Antiseptic Soap and keep it between sheets of blotting paper sprinkled with antiseptic talcum powder

To Prepare the Floor of the Cap for a Porcelain Crown.

Puncture the floor of the cap from the root side with a pointed, three-cornered instrument. A puncture thus made turns up a bur on the porcelain side, which becomes firmly imbedded in the body of the piece and is fully as retentive as a headed pin in the facing.—*L. A. Arnold, Dental Review.*

As it is sometimes difficult to locate just exactly on the plate the irritated spot, place with a spatula a little wet whiting on the spot and put the plate in position and it is readily seen.—*Dr. L. P. Haskell, Dentist's Mag.*

The best clasp for a partial lower vulcanite plate is made of 18 gage gold wire, doubled, but not close, fitted to the tooth on the buccal and lingual side, and the ends turned at right angles and vulcanized to the plate.—*Dr. L. P. Haskell, Dentist's Mag.*

Protection for Facings.—To prevent "etching" of facings when using 20 per cent platinum solder, paint the surface with a creamy solution of carbonate of magnesium before investing. —*A. E. Matteson, Dental Review.*

To Soften Calculus.—Before removing salivary or serumal calculus it will be found advantageous to apply tincture of iodine. A few treatments of this will tend to disintegrate the deposit and thus facilitate its removal.—*E. M. S. Fernandez, Review.*

Retention of Dentures.—If possible two roots should be left in each jaw and utilized to support the plate. A satisfactory method is to fit each such root with a gold cap and tube, into which fit a pin attached to the plate. The stability which even one root so treated will give to an entire denture is surprising.—*Wm. M. Gabriel, Dental Record.*

Icnthyol for Inflammotory and Septic Conditions.—Ichthyol is a most valuable substance to reduce inflammatory swelling and in septic conditions affecting the skin. Its disagreeable odor is very distressing to many patients, but it can be concealed by the addition of oil of citronella, 20 minims to the ounce of ointment. Oil of roses also does well, but is very expensive.—*Med. Standard.*

Burnished Gold Fillings.—*Keep* your burnisher blued; that is, you want to burnish with a blued surface. If it gets bright, gold will stick to the burnisher and you cannot burnish well. Heat the gold burnisher red-hot and allow it to cool off. —*Walter J. Brigham, International Dental Journal.*

To Remove an Impression.—When taking an impression for full superior denture, if it is found difficult to dislodge, have the patient close the lips and blow real hard so as to distend the cheeks and the impression will drop down, no matter how tight it may have been.—*R. C. Traynham, Practical Dental Journal.*

In arranging a lower set commence with the second bicuspids, as four-fifths of the lower anterior teeth are too wide for the uppers, as it is essential that the bicuspids properly occlude, then use teeth just wide enough for the space between the bicuspids.—*Dr. L. P. Haskell, Dentist's Mag.*

Backing for Facings.—A very satisfactory backing for facings is obtained by using 1-1,000 platinum, doubled and burnished to the facing. Its advantages are: Adaptability to the facing, less likelihood of burning the backing while soldering, and reflection of color through facing.—*Henry C. Lee, Dental Review.*

Silver Nitrate; Caution.—The use of silver nitrate in the mouth requires caution. In case of accident its action upon soft tissue can be almost instantaneously checked by promptly applying sodium chlorid, thus forming the insoluble silver chlorid. Extemporaneously, nitrate of silver may be prepared by dipping a silver wire into nitric acid and thus applying it to the tissues. —*Hermann Prinz, Dental Digest.*

To Prevent Saliva and Tooth-Polishing Powders from Working Back into the Handpiece.—One of the cup-shaped rubber disks used for polishing purposes, removed and placed on the shank of the instrument, obviates this difficulty; a ring of wire soldered on the shank will prevent the disk from working back toward the point of the instrument.—*Chas. O. Kimball, Internat. Dental Journal.*

Bubbles in Porcelain.—I believe the reason I do not have bubbles is that in mixing the body I spatulate it with a great deal of force, mix and incorporate it thoroughly, grinding it down with a strong spatula, and when it comes to settling the body, settle it vigorously in one direction. So many when they have a crown or inlay upon a vise, or otherwise, will settle the body in one direction; then they will turn it over and unsettle it again, and rasp it up; changing the original settling of that body until—why shouldn't there be bubbles?—*J. D. Patterson, Western Dental Journal.*

Protecting Porcelain Surfaces for Solder Work:—If porcelain surfaces in bridgework are coated with sandarac varnish before investing, and care is taken to have the investment thoroughly heated before beginning soldering, it will result in little or no cracking or checking of the porcelain.—*Henry C. Lee, Review.*

A Combination Filling.—In the use of soft gold built into a layer of soft cement, followed by cohesive foil and, when subject to abrasion, finishing with gold platinum foil, the good qualities of each ingredient are magnified and the faults of each minimized. The cement is adhesive, the soft gold gives close adaptation, the cohesive gold resists lateral stress in contour, and the alloy of gold and platinum resists abrasion.—*Clyde Davis, Western Dental Journal.*

Why Porcelain Changes Color in Fusing.—The changing of the color in the mass, to my mind, is due more to the movement in the molecules in the mass than it is to the burning out, and when the temperature is raised the molecules are changed and the coloring matter sinks or gravitates toward the center under high pressure, the tendency of the mass being to globulate. This is true of changes that take place in the inorganic world. —*G. W. Cook, Digest.*

Texas State Dental Association.—At a meeting held in June the folowing officers for the ensuing year were elected: President, Dr. R. D. Griffis of Paris; first vice-president, Dr. A. A. Dyer of Galveston; second vice-president, Dr. Charles H. Edge of Houston; secretary and treasurer, Dr. G. Waller Staples of Dallas; curator of museum, Dr. A. F. Sonntag, of Waco. Executive committee: Dr. Charles L. Watson, Mexia; D. J. W. Combs, New Braunfels.

Pyorrhoea Alveolaris: Common Salt.—In a case which had resisted treatment for a great many years, after the loss of many teeth a cure was affected through the use of common salt worked down into the pockets several times a day, salt being also used on the toothbrush. The gums became solid and healthy and recession stopped. The loose teeth became so firm that ,they were used as supports for a bridge.—*E. H. Allen Dental Review.*

Prof. W. D. Miller to Become Dean of the Dental Department, University of Michigan.—As some of our readers may know from newspaper reports, Professor Miller will return to America, and has been called to the chair of dental histology and pathology in the University of Michigan, and to be the dean of the dental department. This will be welcome news to his friends here and the friends of the University of Michigan.

It is, however, a more significant matter for the entire profession.—*Register.*

"*Hand-me-down*" *Crowns*.—Not one thing to-day is doing crown and bridge work, that intricate union of operating room and laboratory, greater harm than these "ready-made," "hand-me-down" crown methods; shells made on composite sizes like shoes; seamless systems no end; "fit guaranteed and crown on in twenty minutes;" crowns constructed from plaster casts shipped to the wholesale laboratory man who advertises: "Let us do your work while you sleep, six thousand bridges made last year, see our testimonials."—*Clarence J. Grieves, Dental Summary.*

Narcotile Anesthesia.—The beauty about narcotile anesthesia is its pleasantness. Patients are insensible to pain long before they are past talking. I can go ahead and operate, the patient being almost entirely conscious, but feeling slight or no pain. I have given narcotile and removed temporary abscessed teeth for almost babies who would find no objection save that "that stuff made their ears roar." The patient always recovers completely in about five minutes, and there are no after-effects. —*Dr. W. H. Reaben, Practical Dental Journal.*

A Hickory Spatula.—The glaze of a glass cement slab being removed, giving a slightly roughened surface. the fine fibre of a hickory spatula permits a mill-stone grind to the mixture of powder and liquid, insuring the breaking apart and turning over and around of all cement particles, giving a more even mixture and securing a more perfect chemical union, with no discoloration.—*Dr. D. R. Phillips, Northwestern.*

Dentist Loses an Eye from Tooth Infection.—Dr. Richards of Glamorganshire, England, extracted a child's tooth and held it up by forceps to show to the mother. A piece splintered off the tooth and struck the doctor's eye. A day or so afterward there were pains in the eye. On examination it was found that a small wound had been inflicted, and, owing to the splinter being decayed, the eye was poisoned. The eye had to be removed, its sight having completely gone.—*Summary.*

Georgia State Dental Society.—At a meeting held in June the following officers for the ensuing year were elected: President ,William Crenshaw, of Atlanta; first vice-president, T. C. Gibson, of Forsyth; second vice-president, C. P. Davis, of Americus; corresponding secretary, J. H. McNeil, of Athens; recording secretary, Delos Hill, of Atlanta; treasurer, H. R. Jewett. of Atlanta; journal editor, H. H. Johnson, of Macon; executive committee, W. C. Miller, of Augusta; W. E. Bugg, of Madison; W. M. Zeirkle, of Atlanta; E. A. Tigner, of Milledgeville; R. Holmes, of Macon; H. H. Johnson, of Macon.

Richmond Metal.—This metal offers the great advantages of very low fusing point—160 degrees, fifty-two below boiling water, enabling you to run a model over a gutta-percha impresion. The formula is as follows: Tin, 20 parts by weight; lead, 19 parts by weight; cadmium, 13 parts by weight; bismuth, 48 parts by weight. In swaging a matrix take impression of cavity with gutta-percha, invest in plaster. It is not necessary to wait for plaster to thoroughly harden or dry; two minutes is ample time when you can run the Richmond metal, giving a metal die on which to swage a matrix.—*J. P. Root, Kansas City Dental Journal.*

Orthoform for Sore Gums.—Give your patient who has had a new plate made a small envelope with a little orthoform in it to use on the plate where it makes the gums sore. This will remove the soreness. Make it a point to see such cases every day for a few days. A little trimming at the right time saves much discomfort and many sore mouths. If a plate tips loose at one side, examine the occlusion on the opposite side and grind the buccal cusps until the lingual cusps occlude the harder. On either side have the lingual cusps occluding slightly harder than the buccal in platework. Do not put teeth beyond ridge if it can be avoided.—*Dr. H. W. McMillan, Tri-State.*

Selecting Teeth for Dentures.—I have two classes of individuals for whom I select colors—the brunette and the blonde. The brunette always has a blue-white tooth, and the blonde a yellow-white tooth—extremely so. You can confirm this with your shade guides when these types of individuals appear in your office. In patients who are neither brunettes nor blondes you will find that one type or the other is predomiating, and you can grade the shades according to the predominating type —*E. J. Perry, Review.*

Skiagraphs as an Aid in Crowning Roots.—Drilling into roots for the purpose of inserting posts for crowns is most treacherous work. Where advisable, we drill a certain distance only, insert a post, and skiagraph and then we learn whether or not we are drilling in the right direction or are dangerously near the apex or side. Again, whether or not a root is sufficiently strong to warrant crowning is sometimes a matter of doubt, and under these circumstances we skiagraph the case, and upon the results shown we base our conclusions.—*C. Edmund Kells, Jr., Dental Review.*

Fusing Porcelain.—In studying fused porcelain under the microscope, I observed that those porcelains which were fused at a lower temperature for a long time, presented more homogeneous and more highly glazed surfaces, while the surfaces of porcelains which were fused at a high temperature for a short time appeared more granular. The cubes were then fractured and the inner portion examined under the microscope. I invariably found that those porcelains which were fused for a long time at a lower temperature were more homogeneous in texture. —*J. Q. Byram, Review.*

Square-end Bur in a Narrow Fissure.—The worst thing I know of in the narrow fissure is the round bottom to the cavity as made with a round bur. In such a cavity your gold will roll and twist, and refuse to lie still. No wonder it is difficult. Take a bur with a square end and cut the fissures leaving square angles along the pulpal wall, and you have no need to build up between the cusps. The gold will stay where you put it. It is then comparatively easy to make perfect margins. One of the secrets of bad fillings has been the making of cavities where the gold will roll; but if you make sharp angles in your cavities you ought not to have any difficulty.—*G. V. Black, Dental Review.*

Excess in Eating.—A hint to those who may thoughtlessly at some time or other indulge in excess eating. If this indiscretion is committed, especially in highly seasoned things with rich sauces, a draught of cold water, acidulated with lemon juice or sulphuric acid, will take off the sense of weight at the stomach and assist the digestite process by moderating the alimentary fermentation. In this connection one may also say that one of the best remedies for obesity, and an exceedingly simple one, is to eat only one thing at a meal. It does not matter greatly what one thing is, whether it is any one kind of fruit or any one kind of grain, but the prescription is: eat one article, and one article only, at one meal.—*Med. Fortnightly.*

Colored Arsenic Mixture.—Dr. Black long ago suggested that we should mix our arsenic with sufficient lampblack so that when placed within the tooth we could see plainly exactly where it lay, and detect any particles that might be disposed to crawl out of the cavity, also upon removal we could be sure of completeness. Anyone who will take the trouble to put up, or order from the druggist, a mixture of arsenic, morphine and cocaine, with lampblack sufficient to make a dull black, and will use this mixture for a time would never think of doing without it.—*Garrett Newkirk, Review.*

Method of Setting Logan Crown.—Prepare the root in the usual manner, and grind the crown to an approximate fit. Mix a small amount of Ascher's Artificial Enamel and place on the base of the crown. Slip over the pin a disk of very thin aluminum or tinfoil and force the crown to place. Remove and lay aside till the "enamel" thoroughly hardens, and with suitable disks, well oiled with vaseline, trim to the line on the foil indicating the periphery of the root. Remove the foil and set in the usual manner. This gives as near a perfect fit as can be made.—*Dr. Mark Hayter, Review.*

Charges.—A dentist should charge for everything he does, even if the fee is only 25 or 50 cents. In the course of a year he does a host of small operations which, with a small fee for each, would amount to quite an item in the course of a year; and at the same time would impress the patient with the fact that time and brains are too valuable to be given away these days. Moreover, patients expect to pay. A small charge should be made for examination and consultation, as these require time, and only one who is qualified can do these tihngs. And don't be afraid that your fee for some painstaking operation is too large. How often have we all had the experience of a pleased patient saying: "Well, doctor, that is better than I expected. I thought it would be twice as much as that."—*Dr. P St. C. Smith, Dom. Jour.*

Fusing Porcelain.—The following deductions are from experiments:

1. That porcelain has no definite fusing point.

2. By prolonging the time of exposure to heat a thoroughly fused porcelain may be obtained at a comparatively low temperature.

3. That porcelains fused at a lower temperature for a long time will maintain their characteristic color.

4. That low--fusing porcelains can be made of high-fusing porcelains by repeated fusing and grinding.

5. If a piece of porcelain is thoroughly fused and more porcelain added and fused, the first layer will be slightly over-fused.

6. That porcelains containing a large percentage of flux are affected more by bubbles than those that are more nearly composed of the basal ingredients.—*J. Q. Byram, Review.*

A Universal Appliance.—The expansion arch with anchor clamp bands comes the nearest to a universal regulating appliance of anything known. It can be made to expand the arch, contract the same, rotate teeth, elongate them, depress them in their sockets, "jump the bite" either forward or backward, expand the arch in one region and contract it in another at the same time, and do all the above with the greatest efficiency and comfort, requiring a minimum of attention from the operator. But little else is needed for any case if the operator studies to use the force at his command in this apparatus.—*Western Dental Journal.*

Putrescent Canals.—I have been using Dentalone, with the best of results, in putrescent canals, in this way: Mix a little Dentalone with iodoform. The old way was to mix iodoform with beechwood creosote, which does the work fairly well, but gives off a very disagreeable odor to the dentist and his patient. With the use of Dentalone the odor is greatly modified. I

think it does the work better than creosote, and I have set the creosote bottle aside and Dentalone has taken its place for good. I would suggest that my brother practitioners try Dentalone for results.—*Dr. W. P. Brinton, Era.*

Taking the Bite.—My way of taking the bite is as follows: Put your patient in a comfortable position just as when you wish to converse with him. Prepare a trial plate in ordinary manner with a piece of warm wax upon it. At the heel of the plate put a clutch made of wax. Tell your patient to put the tip of the tongue within this clutch and close the lips. The patient is unable to throw the lower jaw forward or sideways. The reason I ask them to close the lips is because, when a patient needs a full upper and lower set of artificial teeth they have no teeth to close, and by closing their lips they are not so apt to protrude the lower jaw in case they lift the tongue out of the clutch.—*Dr. R. Kessel, Items.*

Treatment of Children in the Office.—I encourage parents to bring their children to the office, from four years and upward. I get acquainted with the little felows, ask them if they have teeth, too, and how many, inviting the mother to take the chair with the little one on her lap, let them see the mouth mirror, ask them to look in it; then I take the pliers with a pellet of cotton and pass over the teeth, give him a little box or bottle, invite him to come again, and have made a start that will win to myself a well--behaved patient.

In filling the teeth of children I employ any material I can best adapt in a rapid way. Oxyphosphate of copper is my pet. Gutta percha is almost as difficult to introduce as gold, unless the cavity can be thoroughly dried. It pays to treat abscess conditions in children's teeth. I am using with good results a combination of a 50 per cent solution of tricresol and 40 per cent solution of formaldehyde, equal parts. This may be sealed in at almost any stage of putrescence.—*F. H. McIntosh, Review.*

Making an Inlay Matrix.—My own mode of procedure is as follows: First press the metal, gold or platinum, as the case may be, to the floor of the cavity with spunk or cotton, bending the protruding metal parallel to the walls.

Then replace the spunk with guncamphor, then with rotary burnisher direct, or right angle, as large as will go well into the cavity; force the metal up to the cavity walls; remove and anneal; replace in cavity, pack not over full with camphor, bend protruding metal back over edges of cavity, not close, but sufficient to enable you to draw a strip of linen over the tooth, covering camphor and metal, holding linen just tight enough to retain metal and camphor in place. With same burnisher force metal well up to walls all around, finishing with a burnisher that is small enough to go into all angles or grooves.

Never allow burnisher to touch the metal. Remove and after burning out the camphor, fuse enough porcelain in the matrix to cover floor, or enough to fill cavity one-third, then replace in cavity. Fill with camphor, replace the linen strip as before, only draw it and hold *tight* to place. Then with small rotary burnisher burnish over the edges thoroughly, being careful as before to not permit the burnisher to touch the matrix metal.

With a little practice you can in this way make a perfectly accurate fitting matrix with less puncturing or tearing and in less time than I am able to do in any other way known to me. The same procedure can be followed in a model.—*Dr. J. R. Callahan, Review.*

MATRIX FOR PACKING PORCELAIN.—As we all know, the more dense we can pack the body the less shrinkage. In building up a large molar crown to be finished with one baking, I have been pleased with the following experiment: Having the pins gripped firmly in the proper holder, I cut a strip of sheet steel —of the thinnest sort, such as we use for matrices—and pinched this together around the cap with a small screw vise. Then I oiled the inner surface of the matrix to prevent the porcelain from adhering. I was then able to pack the body of the crown very quickly and firmly, absorbing the moisture that was pressed to the surface from time to time with blotting paper. I built high enough to allow for prominent cusps, then released the vise hold upon the matrix. The spring steel let go, leaving a fine general contour and a smooth surface. Then, after having cut away enough of the plaster teeth adjoining to allow about one-sixth for shrinkage, the tooth to be was placed in the articulator and cusps carved to shape. Any required special forms of contour were added with a brush, I am sure that I got less shrinkage and a considerable saving of time by this method.

I may add here that it seems to me that I get a good final surface with soft spunk, dry or slightly moist, upon the nearly dry surface of the body.—*Garrett Newkirk, Review.*

